# Conflating the map with the territory: Challenges for evidence syntheses on homicide in a global context

**DOI:** 10.1177/02685809251336694

**Published:** 2025-05-21

**Authors:** Elizabeth A Cook

**Affiliations:** Violence and Society Centre, City St George’s, University of London, UK

**Keywords:** Global, homicide, inequalitiesInequalities, knowledge production, sex/gender, homicide, inégalités, mondial, sexe/genre, sociologie de la connaissance, desigualdades, global, homicidio, sexo/género, sociología del conocimiento

## Abstract

Homicide is a global burden that is unequal in risk and distribution. However, evidence required for prevention is currently fragmented across different systems of knowledge production, creating challenges in the form of missing data. Viewed through the sociology of quantification and knowledge production, this article provides methodological and ethical reflections on conducting a global systematic review of sex/gender-disaggregated homicide data. In doing so, it highlights epistemological and ontological differences that risk becoming obscured in global, comparative work on violence. The systematic review consisted of a four-step search strategy: electronic database searches, hand searches of statistics, ministry, and police websites, citation tracking, and email survey of statistics offices. Studies were included if they reported prevalence data on homicide which was sex/gender-disaggregated (by victim/offender relationship, sexual aspects, and/or motivation) by both women *and* men. From 194 WHO-recognised countries, data were available for just under half (*n* = 84). However, there were pronounced differences between countries and regions regarding the availability of data. To avoid conflating the ‘map with the territory’ as others argue, this article follows the call from Dalmer for *critical knowledge synthesis* which builds contestation *in* to systematic review and recognises evidence in a wider (and unequal) system of knowledge production.

## Résumé

L’homicide est un fardeau mondial dont le risque et la répartition sont inégaux. Toutefois, les données nécessaires à sa prévention se trouvent actuellement fragmentées entre différents systèmes de production de connaissances, ce qui pose des problèmes de données manquantes. Sous l’angle de la sociologie de la quantification et de la production de connaissances, cet article apporte des réflexions méthodologiques et éthiques sur la réalisation d’un examen systématique mondial des données sur les homicides, ventilées par sexe/genre. Cela permet de mettre en lumière les différences épistémologiques et ontologiques qui risquent d’être occultées dans les études comparatives mondiales sur la violence. Pour réaliser cet examen systématique, une stratégie de recherche en quatre étapes a été appliquée : recherches dans les bases de données électroniques, recherches manuelles sur les sites web de statistiques, de ministères et de la police, suivi des citations, et enquête par courrier électronique auprès des bureaux de statistiques. Les études ont été incluses si elles présentaient des données sur la prévalence des homicides, ventilées par sexe/genre (en fonction de la relation entre victime et agresseur, des aspects sexuels et/ou du mobile), aussi bien ceux commis par des femmes que par des hommes. Sur les 194 pays reconnus par l’OMS, des données étaient disponibles pour un peu moins de la moitié (n=84). Des différences marquées entre les pays et les régions ont cependant été observées en ce qui concerne la disponibilité des données. Pour éviter de faire l’amalgame entre « la carte et le territoire », comme d’autres le font, ce document suit l’appel de Dalmer (2020) en faveur d’une *synthèse critique des connaissances* qui intègre la contestation dans l’examen systématique et reconnaisse les données probantes *dans* un système plus large (et inégal) de production de connaissances.

## Resumen

El homicidio constituye una carga global con un riesgo y una distribución desiguales. Sin embargo, la evidencia necesaria para su prevención se encuentra actualmente fragmentada entre los diferentes sistemas de producción de conocimiento, lo que genera desafíos en forma de datos faltantes. Desde la perspectiva de la sociología de la cuantificación y de la producción de conocimiento, este artículo ofrece reflexiones metodológicas y éticas sobre la realización de una revisión sistemática global de datos de homicidios desagregados por sexo/género. De este modo, destaca las diferencias epistemológicas y ontológicas que corren el riesgo de quedar ocultas en los estudios comparativos globales sobre la violencia. La revisión sistemática consiste en una estrategia de búsqueda en cuatro pasos: búsquedas en bases de datos electrónicas, búsquedas manuales en páginas web de estadísticas, ministerios y autoridades policiales, seguimiento de citas y una encuesta por correo electrónico a las oficinas de estadística. Los estudios fueron incluidos si reportaban datos de prevalencia de homicidios desagregados por sexo/género (por relación víctima/agresor, aspectos sexuales y/o motivación) tanto cometidos por mujeres como por hombres. De 194 países reconocidos por la OMS, se dispone de datos para casi la mitad (n=84). Sin embargo, se han observado marcadas diferencias entre países y zonas geográficas en cuanto a la disponibilidad de datos. Para evitar confundir el “mapa con el territorio”, como sostienen otros autores, este artículo sigue la llamada de Dalmer (2020) a una *síntesis crítica del conocimiento* que integre la disputa *en* una revisión sistemática y reconozca la evidencia en un sistema más amplio (y desigual) de producción de conocimiento.

## Introduction

Data and evidence on violence are a few of the many core elements necessary for prevention. They inform decision-making by policy makers, provide corroboration for claims-makers, and exist as a means of empowerment for advocates and activists (D’Ignazio, 2024; Lavorgna and Ugwudike, 2021). As new methods and modes of data collection have accelerated, the range of evidence on violence has expanded with contributions not only from social science disciplines adjacent to sociology such as criminology and international relations, but also from the medical sciences, such as epidemiology, population health, and clinical trial research. Indeed, the pluralist nature of knowledge production in this space is a key strength and is widely considered characteristic of the transnational world (Henne and Troshynski, 2013; Franko, 2021). This includes recognising that different social realities exist in different places, that there are different discourses for making sense of a social reality (e.g. ‘patriarchy’ and ‘capitalism’) (Xie, 2021), but also that there are different ways of knowing social problems.

However, how do we begin to make sense of such a vast body of evidence on violence in a transnational context? And how do we make sense in ways that capture both *ontological* difference (i.e. how acts of violence manifest in different social realities) and balance the range of evidence seeking to represent those acts (*epistemological* difference)? This article contributes answers to these questions by providing reflections on a specific form of evidence synthesis – a systematic review – conducted to estimate the proportion of sex/gender-disaggregated homicide by country, region, and globally. A systematic review is a type of literature review defined ‘as a robust, reproducible, structured critical synthesis of existing research’, typically conducted according to set, a priori defined criteria (Munn et al., 2018: 2). Their overarching aim is to identify, appraise, and synthesise evidence on a given topic. However, there has been limited critique of systematic review applications to synthesising evidence on violence and abuse (for an exception, see Schucan Bird et al., 2023) or their positioning as the ‘gold standard’ in evidence synthesis. This article therefore situates systematic reviews as a form of evidence synthesis that take place within a broader ‘ecosystem’ of evidence, critically considering the role of evidence synthesis in knowledge production on homicide in a global context. As Gough et al. (2019: 2) describe, these ecosystems are shaped by relationships and communities of evidence producers and users, different channels and tools of knowledge production, and a broader ‘socio-political context’ in which evidence is generated. In doing so, the article identifies the key challenges of synthesising evidence in a global context, as well offering a reframing towards *critical knowledge synthesis*, as described by Dalmer (2020), as a solution to overcoming these.

The article proceeds in four parts. The first part sets out the theoretical framework that informs the critical reflections and analyses presented in this article. This framework merges insights from scholarship on the sociology of knowledge and quantification in a global, comparative context (e.g. Bhuta et al., 2018; Merry, 2016) and the role of epistemic worlds and systems in generating knowledge and evidence on homicide and femicide (e.g. Mobayed Vega, 2023; Walby, 2023). The second part introduces the role of evidence synthesis, specifically, systematic reviews before summarising the methodology of a global systematic review on sex/gender disaggregated homicide data. The third part provides reflections on the key challenges to synthesising evidence using systematic review organised around two themes. The first concerns how (or whose) social realities are represented in evidence and considers the risks that certain types of evidence are ‘institutionalized in ways that lead to the present inequalities’ between the Global North and South (Abraham and Purkayastha, 2012: 129; Carrington et al., 2016; Franko, 2021; Xie, 2021). The second examines how methodological processes of selection, retrieval, and data extraction create abstractions of the social problems that evidence syntheses seek to engage with. This raises concerns for how meaningful comparisons of violence can be made where the social realities of that violence are likely to be substantively different across time and place. The fourth and final part offers some potential solutions and optimism for meaningfully engaging with violence as a global concern through evidence synthesis, remembering that it is not a question of *whether* context matters, but *how* (Abraham, 2019). In doing so, it engages directly with assumptions regarding systematic review about ‘procedural objectivity’; that following a set of explicit rules can reduce bias (Hammersley, 2001: 545). This section argues for a re-framing and engagement with evidence synthesis as a form of knowledge production. It follows calls from Chambers et al. (2018) and Dalmer (2020) in moving towards *critical knowledge synthesis*, acknowledging that synthesising evidence involves processes of transformation and labour which shape our empirical outputs.

## Theoretical framework

### Situating homicide within the (global) sociology of knowledge

Globally, evidence on homicide is fragmented across a constellation of different systems of knowledge production. In 2021, the United Nations Office on Drugs and Crime (UNODC) *Global Study on Homicide* reported that there were 458,000 victims of homicide, including a total of 81,000 women and girls (19%) (UNODC/UN Women, 2023; UNODC, 2023a). As an indicator, homicide is often applied as a gauge of global inequality and security, typically because of assumptions that it is *more* likely to be registered and prosecuted by police than other forms of crime and *less* prone to reporting errors. However, these data also matter for questions of whose lives (and deaths) are *knowable* (Franko, 2021: 25), and through what methods they can be known.

What we know can be assembled from a range of sources including court records, newspaper articles, fatality reviews, police reports, death certificates, family testimony, social media posts, as well as as biographical narratives, to name a few. While not exhaustive, there are three systems in particular that can be identified which collect, produce, and/or curate quantitative data on homicide: criminal justice, health, and civil society. Within these systems, evidence on homicide is generated according to different definitions and classifications of sex and gender, race, ethnicity, homicide, and responsibility, and are driven by different units of analysis (Walby et al., 2017). For example, in criminal justice, law enforcement data such as police reports or court records will typically focus on the homicide case or perpetrator, driven primarily by a focus on the criminal investigation or prosecution of a suspect (e.g. the UNODC *Global Study on Homicide* as well as the *World Bank World Development Indicators*; see Rogers and Pridemore, 2023). This has tangible implications for our understanding of violence; for example, murder-suicides will not appear in data based on conviction or court records as the perpetrator is deceased, but is a sub-type of homicide known to be perpetrated by males against female partners (Vatnar et al., 2019).

Alternatively, vital statistics retrieved from civil registries in national health systems, such as death certificates, will typically centre on the victim, with the emphasis being on documenting the cause of death (e.g. the World Health Organization (WHO) *Mortality Database* or *Global Health Observatory*). Adminstrative data collection systems such as these represent what might be described as ‘institutionalised’ forms of knowledge production. However, the production of evidence on violence – and its uptake in policy – is also driven significantly by feminist civil society and activism (D’Ignazio et al., 2022). This can be seen in the increasing numbers of femicide observatories (e.g. the Canadian Femicide Observatory for Justice and Accountability), monitors and blogs such as *Counting Dead Women*, as well as femicide review committees which draw insight across a range of sources to provide an in-depth, chronological account of the victim’s death (UNODC, 2023b). While issues in availability, inconsistency and the completeness of data are challenges common to all of these systems (Giesbrecht et al., 2023; UNODC, 2023a; Walklate et al., 2020), each offers a different window onto the same problem.

Evidently, there is a wealth of national and international data on homicide available. The quantification of social problems, as (Bhuta et al., 2018 12) write, ‘invite comparison’. Indeed, comparative research can be useful for making sense of difference: to confirm or challenge existing evidence, to test and revise theory, and to situate our perspectives on change in relation to others. However, comparison also requires a ‘considerable labour of standardisation and commensuration’ (Bhuta et al., 2018: 12). This might include identifying proxies or variables to represent a problem, drawing parameters around a definition, selecting data sources, or deciding how to manage missing data. Espeland and Stevens (1998: 316) summarise this as follows:

Commensuration transforms qualities into quantities, difference into magnitude. It is a way to reduce and simplify disparate information into numbers that can easily be compared. This transformation allows people to quickly grasp, represent, and compare differences.

Commensuration therefore creates a relation between things that seem different. While issues of measurement and value have been central in the natural and social sciences, most scholarship has remained focused on the *accuracy* of what has been quantified rather than quantification as a ‘sociological phenomenon in its own right’ (Espeland and Stevens, 2008: 402). Scholarship has since developed specifically to investigate the sociology of quantification (Mennicken and Espeland, 2019) and studies of global governance and ‘indicator culture’ (Bhuta et al., 2018; Merry, 2016) and which build upon foundations established within the field of science and technology studies (STS) (see Latour and Woolgar, 1979). These works seek to interrogate the processes of quantification, classification and commensuration as objects of sociological study. Espeland and Stevens (1998: 315) elaborate:

We argue that commensuration is no mere technical process but a fundamental feature of social life. Commensuration as a practical task requires enormous organization and discipline that has become largely invisible to us. Commensuration is often so taken for granted that we forget the work it requires and the assumptions that surround its use. It seems natural that things have prices, that temporality is standardized, and that social phenomena can be measured. Our theories presume that we commensurate when choosing and that values can be expressed quantitatively. Commensuration changes the terms of what can be talked about, how we value, and how we treat what we value. It is symbolic, inherently interpretive, deeply political, and too important to be left implicit in sociological work.

Not only does this matter for the process of how data becomes evidence, but also for governance, regulation, and decision-making, that is, how evidence is applied. As others have argued, to regulate and govern, we need to know (Merry, 2016). This involves thinking more than about the objects that emerge (e.g. an indicator, a variable, a classification system) (Bowker and Star, 2000), but how it is made legible in the first place.

### Epistemic cultures, communities and systems

Data and evidence are also shaped by the communities of knowledge production in which they are generated and the rules and structures of governance which they are bound by. As evidence on violence has fragmented, several authors have noted the emergence of specialised disciplines and ‘epistemic worlds’ (Mobayed Vega, 2023) or ‘systems’ (Walby, 2023). These epistemic systems comprise communities of knowledge which hold particular ways of knowing a problem, such as discursive strategies (words, expressions, concepts) through which violence is interpreted (Strauss, 1978) as well as methodological conventions and ways to establish evidence of something (Knorr and Cetina, 1999). Across systems of criminal justice, health, and civil society, a range of actors and agencies work to shape *and be shaped by* the content and types of knowledge that they produce, whether in the form of policies, protocols, commemorative practices, databases, or statistical frameworks.

These systems might also hold their own normative appraisals of quality and institutional agendas by which they are governed (Star and Griesemer, 1989). In addition, these systems of knowledge production are not static: they are fluid, likely to change and re-formulate over time as they grapple with new social problems and renegotiate out or in tension with other systems of knowledge (Abbott, 2001). Mobayed Vega (2023) offers a sophisticated analysis of the concept of ‘epistemic worlds’ in application to femicide specifically. Merging insights from Foucauldian traditions on machineries of knowledge and STS, Mobayed Vega (2023: 19) identifies six epistemic worlds that ‘craft, contest, and negotiate femi(ni)cide’ as a boundary object between numerous actors and communities. These include the *institutional* (e.g. the formal insitutions that can mandate action and decision-making, such as the UN and UN Women), the *social* (e.g. civil society organisations such as activists, artists, and human rights advocates), the *legal* (e.g. domestic and intenational legal frameworks and systems that criminalise femicide), the *criminological* (e.g. police, prosecutors, and solicitors that pursue criminal investigations), the *statistical* (e.g. frameworks such as the International Classification of Crime for Statistical Purposes (ICCS) and International Classification of Diseases (ICD)), and the *theoretical* (such as sociological, feminist, decolonial, cultural, and political theory, e.g. Corradi et al., 2016). These worlds not only construct social problems differently, but also generate different forms of knowledge, and different forms of evidence to qualify or quantify what is known. This influences the data sources that are employed and the definitions used to classify different homicides. This might include, for example, ‘staged homicides’, where perpetrators manipulate the crime scene or material evidence of a murder, which might potentially be misclassified as suicide in police or coronial data (Bitton and Dayan, 2019). It might also include femicide scholarship that has sought to re-define death in femicide as ‘the inability to live’ which captures the everyday conditions that place women under the ‘continual threat of being killed’ (Shalhoub-Kevorkian, 2002: 581).

Seeing social problems such as violence through the sociology of quantification and knowledge production is useful for a few reasons. First, these perspectives support an interrogation of evidence about problems that we might sometimes take for granted. They remind us that numbers are ‘seductive’ and that those practices of data collection, analysis, disaggregation, classification, and curation are not as implicit as is sometimes assumed (Merry, 2016). For example, Rogers and Pridemore (2023) demonstrate that definitions, data sources, and calculation methods may all affect the results of cross-national research on homicide – in terms of the significance of relationships, but also in magnitude and even the *direction* of relationships. Second, these perspectives encourage a critical engagement with ‘taken-for-granted knowledge structures’: that is, systems of knowledge production which are unquestioned and, therefore, so too are their products (Abraham and Purkayastha, 2012: 133). Making comparisons in a transnational context can highlight different social realities, while at the same time, reinforcing the need to de-Westernise sociology: to generate knowledge that represents non-Western social realities and to avoid hierarchies of knowledge production, synthesis and appraisal typically rooted in the Global North (Carrington et al., 2016; Franko, 2021; Xie, 2021). Third and finally, questioning ‘taken-for-granted knowledge structures’ therefore also compels us to question taken-for-granted *methodologies*. This involves questioning conventional steps of, for example, a systematic review to consider how conventional practice under-represent particular types of evidence or knowledge (such as peer-reviewed publications taking precedent over grey literature). The next section addresses evidence synthesis, specifically, systematic reviews, in this light.

## Methods

### What is evidence synthesis?

Accompanying the rise of evidence-based practices (EBP) and information sciences in the early 1990s, systematic reviews have become a customary method of evidence synthesis. They are routinely located at the top of the ‘evidence hierarchy’ in evidence-based medicine (EBM) and related disciplines, which ranks evidence based on their level of rigour, and are frequently referred to as the ‘gold standard’ of EBP (Greenhalgh et al., 2018). A systematic review is conducted for the purpose of collecting the evidence relevant to a specific question and making an assessment of its quality according to a set of pre-defined and transparent criteria (Gough et al., 2019). While originating within EBM, systematic reviews have since been applied within public policy, education, management, health and social care, and criminology.

Translating the principles and methods of systematic review between disciplines has raised several questions, not least regarding how to adapt these methodologies across disciplinary boundaries, but also how to tailor them to respond to synthesising evidence on violence and abuse specifically. Schucan Bird et al. (2023: 1056) highlight several challenges that the field of domestic violence and abuse (DVA) presents for conducting systematic reviews: for example, the ‘absence of standardised measures’ and outcomes, different definitions of DVA (e.g. physical, sexual, emotional), and that study populations typically consist of ‘relatively homogeneous population groups (predominantly heterosexual, female, white, adult samples)’ from specific geographical, often urban, areas. However, conducting systematic reviews on violence and abuse also presents an opportunity to unsettle and reflect on assumptions that have remained implicit in the process of evidence synthesis thus far. The next section presents a summary of the systematic review which forms the basis for these reflections.

### The current study

This article reflects on a systematic review seeking to estimate the prevalence of sex/gender-disaggregated homicide by country, by region, and globally. The review was conducted as part of a UK Prevention Research Partnership-funded consortium, VISION (MR/V049879/1), to improve the measurement of violence as a cause of health inequalities. Acknowledging that violence is a complex problem, a team science approach was applied; an interdisciplinary approach which brings together and leverages knowledge, methods and skills from diverse disciplines to combine their strengths. The core team involved a legal scholar, a methodologist, primary care researcher, an international relations scholar, and a sociologist/criminologist (the author) who were all funded members of the consortium, as well as drawing upon the expertise of librarians and statisticians. This was the author’s first experience of conducting a systematic review and therefore consisted of an iterative process of accessing online training materials and manuals (e.g. the *Cochrane Handbook*, see Higgins et al., 2023), weekly supervision and team troubleshooting workshops with experienced methodologists, and essentially ‘learning by doing’. Various consortium members were therefore involved in the screening, extraction, quality assessment, analysis, and writing stages of the systematic review. However, this article provides reflections from the perspective of a sociologist/criminologist.

The objective of the review was to provide an update of Stöckl et al.’s (2013) systematic review which estimated the national, regional and global prevalence of intimate partner homicide. In addition, the current study sought to *expand* the number of sex/gender dimensions collected. Therefore, applying Walby et al.’s (2017) framework, we also sought prevalence data on homicides by other types of victim/perpetrator relationship (including homicide by a family relation (including parent, child, and other), acquaintances, and strangers), whether there were any sexual aspects to the homicide and, if possible, motivations for homicide. The author led each stage of the review with support from fellow team members who were responsible for second screening at abstract and full-text stage, checking data extraction and quality assessment, and conducting data analysis. The author also benefitted from generous consultation and expertise from one of the original review authors. These stages included formulating the research question and determining the scope, developing a search strategy and identifying databases, registering the study protocol, setting inclusion and exclusion criteria, screening the searches, data extraction, quality appraisal, analysis, and, finally, the presentation of results.

The protocol for the review was registered on an international prospective register of systematic reviews (Protocol I.D.: CRD42021268712). The review consisted of a four-step search strategy, including electronic searches of five databases (MEDLINE, Global Health, Embase, Social Policy and Practice, and Web of Science), hand searches of statistics, ministry, and police office websites, backward and forward citation tracking (conducted in June and July 2022), and an email survey of national statistics offices. No restrictions were placed on the study language or settings (i.e. location). A report was eligible for inclusion if they reported prevalence data on homicide which was sex/gender-disaggregated (i.e. reporting the victim/offender relationship, sexual aspects, and/or motivation) by both women *and* men, they represented one of the 194 WHO-recognised countries (as per Stöckl et al., 2013), and was published after the 1 January 1990. Initial database searches were conducted in September 2021, and were updated in November 2023 and November 2024. Two concept clusters (‘homicide’ and ‘gender’) were combined, linked by a Boolean operator. Each concept was represented by multiple search terms: for example, homicide was represented by terms such as ‘murder’, ‘killing’, ‘femi(ni)cide’, ‘feminicidio’, ‘patricide’, ‘matricide’. This search string was based upon the original set of search terms used in Stöckl et al. (2013) but updated and expanded to reflects relationships outside the intimate context.

**Figure 1.**
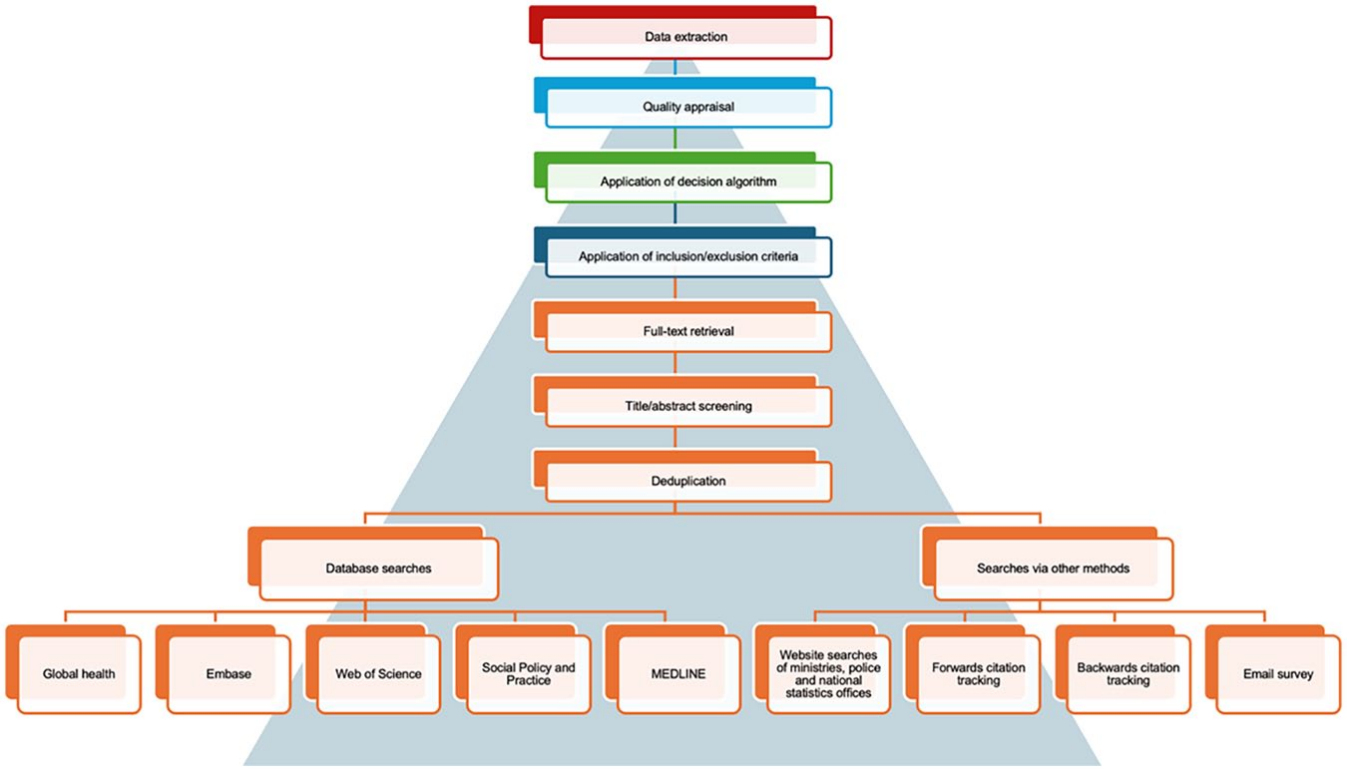
Summary of stages of the systematic review.

This strategy returned a total of 16,785 records, which reduced to a total number of 10,059 for title and abstract screening once duplicates and reports where full texts could not be retrieved had been removed. After completing title and abstract screening, a total of 1840 full texts were screened to assess whether they met the inclusion criteria before applying a decision algorithm to identify one study for *each* country and *each* outcome. A decision algorithm is a process applied in evidence synthesis to select the best evidence according to a specific outcome when you have multiple sources of evidence available. We employed a decision algorithm in this systematic review in keeping with Stöckl et al.’s (2013) original review, prioritising data that was first, demographically representative, second, with more complete information on the selected outcome, third, using more inclusive definitions, and finally, covering more years and that are more recent. Following quality appraisal, the final phase of data extraction consisted of re-reading each study according to a framework agreed upon by myself and in iteration with other team members including items such as outcomes, populations, units of analysis, and study design. A flow diagram summarising these key stages is provided in Figure 1.

## Challenges

The reflections offered here are partly ethnographic, from the point of view of the author and the everyday practice of conducting a systematic review, and partly a comment on the epistemological tensions and opportunities of evidence synthesis on homicide in a global context. These reflections are divided into two themes: first, the *under-representation* of evidence from certain countries and regions and, second, the risks of *mis-representing* experiences of violence through what Moreira (2007) describes as ‘disentangling’ evidence on violence from one context and ‘re-entangling’ it as part of another, such that violence likely to be substantively different across time and place are conflated.

### Under-representation: ‘Gaps’ in global evidence and who they represent

At the time of writing^1^, there were a total of 465 unique reports, corresponding to 419 unique studies, that were included in this systematic review. Overall, there were a total of 84 countries, represented by 465 unique reports, with eligible data for inclusion in the systematic review. However, echoing previous critiques from perspectives of global health (Kumar et al., 2022) as well as sociology (Connell et al., 2017), there was a demonstrable skew in the reports eligible for inclusion in the systematic review by country and region^2^. This can be seen first in *Table 1* which presents the included reports (and therefore represented in the review) by six WHO-classified regions (see WHO, n.d., for a full list). This table describes the representation of regions not the volume of output for each region. Of countries with eligible data, 44% (*n* = 37) were from the European Region with a further 23% (*n* = 19) from within the Region of the Americas. In comparison, only 5% (*n* = 4) of the represented countries were from the South-East Asia Region, while 7% (*n* = 6) were from the Eastern Mediterranean Region. These trends also remain true if we turn to how countries are represented in terms of proportions of evidence. For example, the United States contributed 92 out of the 435 reports with eligible data (21.1%), followed by 37 from India (8.5%), 35 from the United Kingdom (8%), 28 from Canada (6.4%), and 18 from Australia (4.1%). When mapped onto the most recent World Bank (n.d.)^3^ classifications of regions by income level, a similar picture emerges (see *Table 2*). From the four groups classified by income, a significant proportion of countries with eligible data are provided from the High Income group (44%, *n* = 37), with a further 29% (*n* = 24) from the Upper Middle group. Only 5% of countries came from the Low Income group (*n* = 4). Therefore, there are significant disparities in terms of geographical coverage and by income group, with a majority (72%) of evidence contributed from higher or upper middle income countries.

**Table 1.** Breakdown of eligible data by WHO-classified geographical region.

Geographical region (WHO, n.d.)	Countries with any eligible data	
	%	***n***
African Region	12	10
Eastern Mediterranean Region	7	6
Region of the Americas	23	19
South-East Asia Region	5	4
European Region	44	37
Western Pacific Region	10	8
**Total**	**100**	**84**

Due to rounding, percentages add up to more than 100.

**Table 2.** Countries with included data by World Bank Income Classification Group.

Income classification group (World Bank, n.d.)	Countries with any eligible data	
	%	*n*
Low Income	5	4
Lower Middle	21	18
Upper Middle	29	24
High	44	37
Unclassified^3^	1	1
**Total**	**100**	**84**

However, these evidence ‘gaps’ cannot be seen in isolation from the structural contexts in which these data are produced: how knowledge production is shaped or constrained by existing research funding flows (Kumar et al., 2022), whether data infrastructures are supported in meaningful and sustained ways (Mago and Dartnall, 2021), and how power imbalances are (or are not) managed in a global economy of knowledge (Connell et al., 2017). As this systematic review demonstrates, there are significant disparities both in terms of countries with *any* eligible data for inclusion, but also in the *proportion* of eligible data each country contributes. There is an over-representation of richer countries predominantly from the Global North (e.g. the United States, the United Kingdom, Canada), an under-representation of poorer countries providing sex/gender disaggregated data, as well as complete *non*-representation for roughly half of the countries in the world.

These disparities cannot be understood without looking at the the *infrastructures* of knowledge production themselves. Even perhaps to refer to evidence ‘gaps’ leaves assumptions about what we know and how we know it unquestioned: a gap in one discipline or system might not be so in another, and a gap in evidence might depend on what type of evidence you are looking for. For example, where violence occurring in adolescent intimate relationships is not recognised by law enforcement systems unless over the age of 16, but may be captured in child protection systems in terms of ‘witnessing’ domestic abuse (Weir et al., 2025). Speaking of ‘gaps’, as Sandberg and Alvesson (2011: 25) argue, is ‘more likely to reinforce or moderately revise, rather than challenge, already influential theories’ of knowledge, leaving very little room for innovation or alternative ways of knowing.

### Mis-representation: The costs of breadth over depth

Numerous international protocols have been introduced placing obligations on governments and agencies to monitor progress on gender equality and the elimination of violence against women and girls^4^ (García-Moreno and Amin, 2019). These obligations require data not only to be disaggregated by sex and other inequalities (such as age and ethnicity) but also to identify areas that women and men experience aspects of their lives differently, such as work and employment, education, health, and violence. In other words, gender analyses require more than sex-disaggregated data: they require sensitivity to how patterns of social behaviour and the data that they generate reflect gendered dynamics, relationships, and inequalities in society.

To be eligible for inclusion in this systematic review, a report needed to provide data on both women *and* men (applying Walby et al., 2017). This decision also ensured that the number of records returned from the database search was manageable and feasible (although restricting the search in this way still resulted in a large corpus of data – over 8000 records were returned, and their abstracts screened). However, what was lost in the course of this decision, and which only became apparent in the quality appraisal stage, was any evidence that could effectively speak to violence as shaped by intersecting inequalities. The systematic review’s primary outcome of interest was sex/gender disaggregated homicide, but only a handful of studies cross-tabulated this with other intersecting forms of marginalisation such as race and ethnicity, migration status, age, disability, or gender identity. For example, only 11 of the 84 countries represented in the review provided sex/gender disaggregated homicide cross-tabulated by race or ethnicity.

As studies could only be included if they reported data on women *and* men, a vast and rich body of evidence on femicide/feminicide was not included which could have provided information on indicators of gendered motivation, circumstances, victim and perpetrator histories, and relationship dynamics, as well as those intersectional inequalities mentioned above (Boonzaier, 2023; Dawson and Carrigan, 2021; Mobayed Vega and Gargiulo, 2024). The knock on effect of this decision was that, as much femicide data is produced and driven by data activists, forms of evidence from these systems of knowledge production were not represented in the review. This includes knowledge produced within femicide observatories, femicide review committees, and activist-driven counterdata efforts, and shaped extensively by social movements and institutions based within Latin America (D’Ignazio et al., 2022; Mobayed Vega, 2023). This perhaps speaks less to an inequality in knowledge production, and more about what can be lost in pursuit of methodological convention.

There is an extensive literature that seeks to improve the practice of systematic review providing reporting guidelines, quality appraisal toolkits, and handbooks outlining best practice at every stage of the review process. Indeed, a key enterprise of the systematic review methodology is the identification, selection, and extraction of data from existing research according to a specific protocol (Sandelowski, 2008). The process of data extraction typically involves reading the text of a report according to a structured template which has been designed by the reviewer (or team of reviewers) with the review protocol and primary outcomes of interest in mind. This resembles Espeland and Stevens’ (1998: 317) description of the commensuration process as partly a system ‘for discarding information and organizing what remains into new forms’. The data extraction template for this study included items such as the Study and Report I.D., Country, Region, Funder, Data Source, Method of Analysis, Description of the Population of Interest, Study Inclusion and Exclusion Criteria, Population of Interest Demographics, Definition of Homicide, Definition of Outcome (such as Victim/Perpetrator Relationship) followed by space to insert the values of each outcome, if available.

However, in doing so, information is abstracted from reports which vary in purpose (e.g. census yearbooks, annual police reports, academic outputs, autopsy studies), using different methods (e.g. observational, case-control or cohort studies), with different objectives (e.g. to provide a country profile of homicide, to look at associations between changes in firearm laws and trends in intimate partner homicide, to examine injury profiles and scales) and are re-qualified as data for a different purpose: to conduct a systematic review to estimate prevalence on sex/gender disaggregated homicide. What is at risk during this process of dis-entanglement and re-qualification is a loss or potential flattening of difference between diverse social realities of violence.

This was particularly evident during the data extraction process for the outcome of homicide motive: despite motive being central to criminal investigations of homicide, there was very little consistency in how categories of motive were recorded and reported between countries. Certain types of motive did reappear in the reports, in particular ‘robbery’ and ‘argument’, although there was often conflation with other homicide motives (e.g. ‘revenge’ was often presented alongside or interchangeably with ‘reckoning’, ‘grudge’, or ‘settlement’, while ‘argument’ appeared with ‘quarrel’, ‘altercation’, ‘dispute’). Categories of motive were routinely left undefined, and it was not clear whether they were mutually exclusive. Conceptually, it is possible one homicide could be categorised as an ‘argument’, that took place within the context of ‘family conflict’ with an acute interest in ‘economic gain’.

Even more, one could argue that it is not ethical, nor methodologically sound, to conflate data on homicides that occur in specific socio-historical, cultural, and political contexts. By reading and extrapolating data as part of a systematic review, not only is there a risk of conflating different social realities of homicide, but unintentionally re-presenting them through a frame for which they were not intended. For example, the term ‘honour’ in relation to the murder of women and girls in Palestine must, as Shalhoub-Kevorkian (2002: 583) argues, be seen within ‘the context of a nationalist struggle’ where violence against women is intimately linked with national honour and historical oppression. Another example can be found in the problem of witchcraft-related killings in Ghana, West Africa, often perpetrated against older women. Adinkrah (2004: 335) explored the intersections between ‘[p]atriarchal attitudes, misogynistic beliefs, and ageist values’ as mediating witchcraft beliefs in Akan communities in Ghana. Without locating the motivations behind homicide within social, political and economic contexts in which they occur, we risk not only losing sight of the different social realities of violence, but providing meaningful explanations of why it occurs.

## What can be done? Towards critical knowledge synthesis

What may be evident from this discussion so far, is that there are typical and prescribed practices of conducting and reporting systematic reviews. There is a strong emphasis on ‘search strategies’, ‘inclusion/exclusion criteria’, ‘eligibility’, ‘retrieval’, ‘risk of bias’, ‘appraisal’ and ‘screening’, ‘selection’, which speak to a process of *disentanglement* consisting, as Moreira (2007: 183) argues, of:

strategies attempt to break the ties between data and the original milieus where they were produced. Attempts at dissociation are accompanied simultaneously by practices of *qualification*. It is a central concern in systematic reviewing that the results produced do not merely reproduce the research examined. Thus, another of reviewers’ main epistemic challenges has to do with the transformation of the data being selected, elicited and abstracted. (Original emphasis)

These strategies of disentanglement involve a process of transformation of data, to extract and disassociate data from one setting and re-place and re-frame it another.

Systematic reviews are often hailed as a ‘gold standard’ for decision-making in health, social, and public policy. They are often portrayed with an air of ‘objectivity’ because they follow a specific protocol, pitted in contrast to (supposedly) unsystematic ‘narrative reviews’ (Greenhalgh et al., 2018). However, as Sandelowski (2008) asks, why does adherence to a protocol make systematic reviews objective, as if the steps we enshrine in a protocol are not choices or products of processes which are inherently *subjective*? Tasks such as reading, writing, and interpreting data take place in all types of research, but become obscured in the claims of ‘objectivity’ that proponents of systematic reviews sometimes make.

Rather, if we view systematic reviews as a contested process of knowledge production and negotiation, we can highlight the ‘systematic review enterprise is an interaction between readers and texts that are read, re-read, re-written, or never read at all’ (Sandelowski, 2008: 108). Viewing systematic reviews as a process, exchange, or a collaboration for knowledge production allows us to question taken-for-granted assumptions about how review questions are formulated, how parameters are drawn around what evidence should be included, and why and who chooses to draw those parameters. In doing so, we can start to de-naturalise those forms of evidence which have become institionalised, and the ways in which they reinforce enduring hierarchies in knowledge structures.

This framing of systematic reviews also influenced the decisions made throughout the study process. For example, when conducting quality appraisal, we produced an adapted version of the Joanna Briggs Institute Systematic Review for Prevalence Studies (JBI, 2020) tool was created to accommodate for estimates of homicide specifically. This included items such as assessing the level of missing data on sex/gender, age, and race or ethnicity of the study subjects. In addition, we decided not to conduct a meta-analysis due to concerns that this would replicate the problem of ‘comparing apples and oranges’, especially considering the variation in types of evidence, sources of data, and range of search strategies by which we identified these sources (Pratt, 2010). Reflecting on the earlier stages of the study, there is perhaps also a case to be made for recording in situ reflections about the decisions made in addition to mandatory recording of the technical rationales.

What this article has done is provide reflections on the implications of these abstract stages of systematic review for how evidence on homicide is produced and re-produced in ways that can gloss over differences within and between social realities. In doing so, it follows calls by Chambers et al. (2018) and Dalmer (2020) to approach reviews as ‘critical knowledge synthesis tools’, whereby researchers acknowledge and reflect upon the social relations, practices, communities, and systems of knowledge production that a review is produced within. It seeks to move on from the positivist origins of systematic reviews, leaning away from hierarchies of evidence and prescribed searching and reporting methods, and towards critical dialogue with reviews as a form of knowledge production in itself.

There are also lessons to be drawn from bringing feminist and intersectional approaches, which have previously ‘revealed the [gendered] epistemological roots of knowledge production’, to evidence synthesis in a global context (Abraham and Purkayastha, 2012: 127). These methods recognise that ‘knowledge is often the result of power, privilege and domination and is never completely objective’ (Barberet, 2022: 307) and that legacies of colonisation still shape ways of knowing (Connell et al., 2017). By bringing these approaches to critical knowledge synthesis, we can explore the potential of *building in* contestation to evidence synthesis: examining processes of standardisation and quality appraisal, unpacking practices of selecting, retrieving, and extracting data from evidence, observing how epistemic communities shape different forms of evidence (*and* how they are synthesised), and tracing the uptake and implementation of certain types of evidence into practice (Abraham and Purkayastha, 2012). In this way, critical knowledge synthesis acknowledges the emotional, social, and practical labour involved in the production of data and evidence on violence.

## Conclusion

Homicide is a global burden that is unequal in its risk and distribution. However, the evidence required as part of a broader effort to prevent homicide is currently fragmented across different systems of knowledge production. What we know can be assembled from a range of sources including mortality data generated from civil registries and vital statistics in the health system, criminal justice data such as offence, arrest, and conviction records, and the increasing number of femicide indices and observatories driven by feminist activists. At the same time, this plurality of data and evidence is a key strength of the field of violence and abuse: as Dourish and Gomez Cruz (2018: 1) write, data ‘do their work in relation to other data’. Together, they can reinforce or challenge assumptions, answer or raise more questions, and shift taken-for-granted ways of knowing social problems.

Evidence syntheses are just one way of trying to make sense of this vast body of evidence in a transnational context. Viewed through sociological work on quantification and epistemic communities (Mobayed Vega, 2023), this article has provided reflections on a global systematic review to establish the prevalence of sex/gender disaggregated homicide by country, region, and globally. Challenges were identified which spoke to issues of under-representation and mis-representation and which raise concerns as to how (or whose) social realities are represented in evidence synthesis. By reflecting on different stages of systematic review (such as the selection, screening, and extraction of data from texts) (Moreira, 2007; Sandelowski, 2008), the risks that this posed for conflating different experiences of violence were unpacked acknowledging that they are likely to be substantively different across time and place. The article concluded in support of calls by Chambers et al. (2018) and Dalmer (2020) to move towards *critical knowledge synthesis* as a way to build *in* contestation and to decenter assumptions of objectivity which has typically accompanied this methodology.

Some might argue that adding more qualitative nuance might undermine the very point of quantification. However, examining processes of quantification, comparison or commensuration are important not only because they encourage accountability (Barberet, 2022), but also because, as Merry (2016: 2) argued, they *can* produce better evidence. In this way, global systematic reviews could avoid the hazards of ‘reducing’ or flattening differences in experiences of violence and move more towards amplification (Dourish and Gomez Cruz (2018), recognising that communities of voices can be empowered in relation to one another.

## Notes

1. While the systematic review is complete, it is possible that database searches will be updated and re-run prior to publication of future outputs; therefore, the figures cited within this article should be treated with caution and as provisional. They are reported here for the purposes of explanation.

2. Discussions of country and region of a report is based upon the population of the study, not the location of the corresponding author.

3. The World Bank reports that Venezuela is temporarily unclassified as of July (2021) (see World Bank, n.d.).

4. For example, the Convention on the Elimination of all Forms of Discrimination Against Women (CEDAW), the Beijing Platform for Action, and the 2030 UN Sustainable Development Agenda, 1994 Inter-American Convention on the Prevention, Punishment, and Eradication of Violence against Women (known as Belém do Pará Convention), the 2011 Council of Europe Istanbul Convention, and the 2003 Maputo Protocol in Africa.
